# Monoallelic *Heb/Tcf12* Deletion Reduces the Requirement for NOTCH1 Hyperactivation in T-Cell Acute Lymphoblastic Leukemia

**DOI:** 10.3389/fimmu.2022.867443

**Published:** 2022-03-24

**Authors:** Diogo F. T. Veiga, Mathieu Tremblay, Bastien Gerby, Sabine Herblot, André Haman, Patrick Gendron, Sébastien Lemieux, Juan Carlos Zúñiga-Pflücker, Josée Hébert, Joseph Paul Cohen, Trang Hoang

**Affiliations:** ^1^Department of Pharmacology and Physiology, Université de Montréal, Institute for Research in Immunology and Cancer, QC, Canada; ^2^Department of Translational Medicine, School of Medical Sciences, University of Campinas, Campinas, Brazil; ^3^Centre de Recherches en Cancérologie de Toulouse (CRCT), Université de Toulouse, Institut National de la Santé et de la Recherche Médicale (INSERM), UMR-1037, Université Toulouse III Paul Sabatier (UPS), Toulouse, France; ^4^Unité de recherche en hémato-oncologie Charles-Bruneau, Centre de Recherche du CHU Sainte-Justine, Montréal, Canada; ^5^Department of Biochemistry and Molecular Medicine, Institute for Research in Immunology and Cancer, Université de Montréal, Montréal, QC, Canada; ^6^Department of Immunology, University of Toronto, and Sunnybrook Research Institute, Toronto, ON, Canada; ^7^Institut universitaire d’hémato-oncologie et de thérapie cellulaire, Hôpital Maisonneuve-Rosemont, Montréal, QC, Canada; ^8^Quebec Leukemia Cell Bank, Centre de recherche de l’Hôpital Maisonneuve-Rosemont, Montréal, QC, Canada; ^9^Department of Medicine, Université de Montréal, Montréal, QC, Canada; ^10^Department of Computer Science and Operations Research, Université de Montréal, Montreal, QC, Canada; ^11^Université de Montréal, Montreal, QC, Canada

**Keywords:** SCL/TAL1, LMO1, HEB/TCF12, E2A/TCF3, NOTCH1, T-cell acute lymphoblastic leukemia, tumor suppressor genes

## Abstract

Early T-cell development is precisely controlled by E proteins, that indistinguishably include HEB/TCF12 and E2A/TCF3 transcription factors, together with NOTCH1 and pre-T cell receptor (TCR) signalling. Importantly, perturbations of early T-cell regulatory networks are implicated in leukemogenesis. NOTCH1 gain of function mutations invariably lead to T-cell acute lymphoblastic leukemia (T-ALL), whereas inhibition of E proteins accelerates leukemogenesis. Thus, NOTCH1, pre-TCR, E2A and HEB functions are intertwined, but how these pathways contribute individually or synergistically to leukemogenesis remain to be documented. To directly address these questions, we leveraged *Cd3e*-deficient mice in which pre-TCR signaling and progression through β-selection is abrogated to dissect and decouple the roles of pre-TCR, NOTCH1, E2A and HEB in SCL/TAL1-induced T-ALL, *via* the use of *Notch1* gain of function transgenic (*Notch1IC*^tg^) and *Tcf12*^+/-^ or *Tcf3*^+/-^ heterozygote mice. As a result, we now provide evidence that both HEB and E2A restrain cell proliferation at the β-selection checkpoint while the clonal expansion of SCL-LMO1-induced pre-leukemic stem cells in T-ALL is uniquely dependent on *Tcf12* gene dosage. At the molecular level, HEB protein levels are decreased *via* proteasomal degradation at the leukemic stage, pointing to a reversible loss of function mechanism. Moreover, in *SCL-LMO1*-induced T-ALL, loss of one *Tcf12* allele is sufficient to bypass pre-TCR signaling which is required for *Notch1* gain of function mutations and for progression to T-ALL. In contrast, *Tcf12* monoallelic deletion does not accelerate *Notch1IC*-induced T-ALL, indicating that *Tcf12* and *Notch1* operate in the same pathway. Finally, we identify a tumor suppressor gene set downstream of HEB, exhibiting significantly lower expression levels in pediatric T-ALL compared to B-ALL and brain cancer samples, the three most frequent pediatric cancers. In summary, our results indicate a tumor suppressor function of HEB/TCF12 in T-ALL to mitigate cell proliferation controlled by NOTCH1 in pre-leukemic stem cells and prevent NOTCH1-driven progression to T-ALL.

## 1 Introduction

Thymocyte reprogramming into self-renewing cells is a mandatory event in T-cell leukemogenesis, induced by aberrantly expressed oncogenic transcription factors (1–6). This initiating event sets a pre-leukemic state, while progression to overt leukemia requires additional collaborating events within pathways that control cell fate in the thymus, to evolve through layers of selective pressure (7–10).

**Figure 1:**
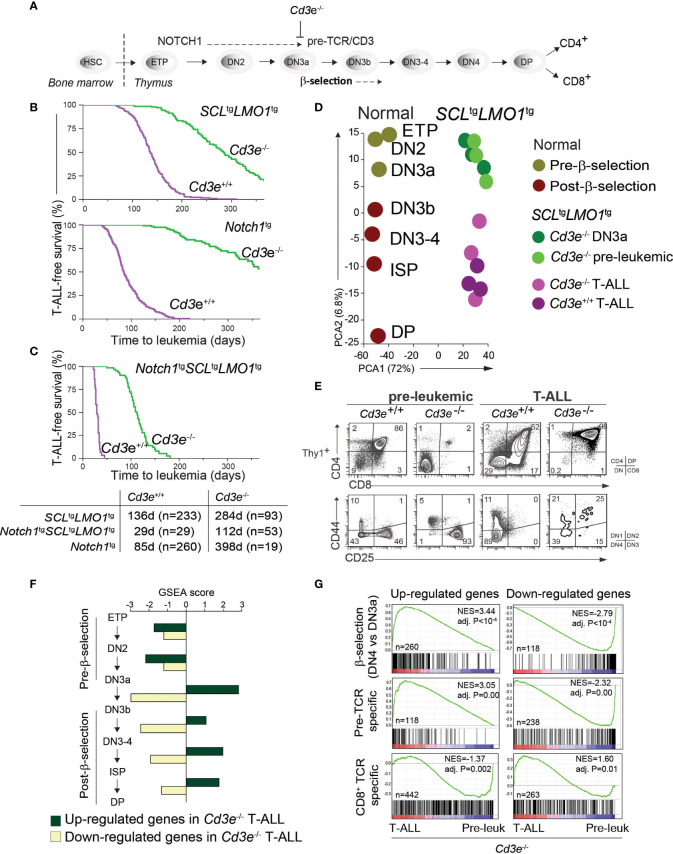
Pre-TCR signaling is functionally important for T-ALL progression. **(A)** Pre-TCR signalling and thymocytes development. **(B, C)** Kaplan-Meyer survival curves comparing disease development in the pre-TCR proficient (*Cd3e^+/+^*) and deficient (*Cd3e^-/-^*) backgrounds in three models of T-ALL. **(D)** Principal component analysis of the transcriptomes of normal thymocyte subsets compared to SCL-LMO1-induced pre-leukemic and leukemic (T-ALL) thymocytes. **(E)** FACS phenotypes of *SCL*^tg^*LMO1^tg^* thymocytes from *Cd3e^+/+^* and *Cd3e^-/-^* backgrounds at pre-leukemic and leukemic stages. **(F)** Gene set enrichment analysis (GSEA) correlates disease progression to stages of thymocyte differentiation. Up-regulated and down-regulated gene sets were computed at each stage of normal thymocyte development from ETP to DP using microarray data from the Immgen project (http://www.immgen.org/), and enrichment was tested during disease progression (pre-leukemia to leukemia). Dark green bars denote enrichment of the up-regulated signature, and yellow bars denote enrichment of down-regulated signatures. **(G)** GSEA analysis of β-selection, pre-TCR specific and CD8+ TCR specific gene signatures during disease progression. Left panels show the enrichment tests for up-regulated gene signatures, and right panels show enrichment of genes decreased by β-selection, pre-TCR and CD8+ TCR.

The first acquisition of full T-lineage identity is marked by successful rearrangement of the T cell receptor (*Tcr*) β locus catalyzed by recombination-activating gene 1 (RAG1) and RAG2 at the CD4/CD8 double negative DN2-DN3 transitional stages (**Figure 1A**). The β-selection checkpoint is controlled by the pre-TCR resulting from the pairing of the successfully rearranged TCR β chain with the invariant pre-Tα chain and the CD3 signaling complex to trigger a burst of cell proliferation and survival, leading to differentiation of DN thymocytes to the CD4^+^CD8^+^ double-positive (DP) stage. Both the pre-TCR and NOTCH1 have obligatory functions at this first checkpoint (11–13). Gain of function mutations of *NOTCH1* are found in more than 55% of childhood T-ALL (14), leading to the well-accepted notion that *NOTCH1* is a major oncogenic event in T-ALL (15–17). The acquisition of *Notch1* mutations in T-ALL absolutely requires pre-TCR/CD3 signaling (4) and involves recombination activating enzymes (RAG1/2) (18). Additionally, the NOTCH1 pathway can also be hyperactive as a consequence of loss of function mutations of FBXW7, the E3 ligase that degrades MYC (19), an essential downstream target of NOTCH1 (20, 21). Nonetheless, the *NOTCH-MYC-FBXW7* triad appears to be genetically unaltered in ~1/3 T-ALL cases, raising the question whether additional genes or pathways may contribute to T-ALL progression.

Comprehensive high throughput sequencing have unravelled the genomic landscape of T-ALL in children (22–24) and adults (25), uncovering a low mutation burden in leukemias compared to solid tumors (26, 27). These studies identified recurring mutations within genes and pathways that control cell fate in thymocytes, confirming the dominant presence of NOTCH1 as a driver mutation. Unlike NOTCH1, oncogenic transcription factors in T-ALL are not mutated but aberrantly expressed in the T lineage driven by chromosomal translocations. These oncogenic transcription factors belong to two families, the basic helix-loop-helix family (SCL/TAL1, TAL2, LYL1) and associated partners (LMO1, LMO2), as well as homeodomain proteins (TLX1, TLX3, HOXA) (reviewed in (28, 29). Transgenic mice in which oncogene expression is driven in the thymus develop T-ALL with variable latency, indicating the necessary acquisition of collaborating events. This is illustrated by the loss of *Bcl11b* function, through transcription repression by the *TLX1* oncogene or through mono-allelic deletion in mouse models (30) and in 9% of human T-ALL (31).

Similar to BCL11B (32), both E2A (E12 and E47 (33)), and HEB (HEBCan and HEBAlt (34)) are essential for the commitment of progenitor thymocytes to the T-cell lineage (35, 36) by governing a gene expression program that is critical for T-cell development [(37), reviewed in (38)] and includes T-cell specific genes such as *Ptcra* and *Cd4* (39, 40), as well as cell cycle genes such as *Cdkn1a* (37, 41). Moreover, E2A is antiproliferative in thymocytes (42–44) and E2A-deficient mice develop T-cell lymphomas (42, 45), indicating that E2A has tumor suppressor functions, much like BCL11B. Nonetheless and unlike *BCL11B*, neither *E2A/TCF3* nor *HEB/TCF12* was found mutated or affected by copy number variations in human T-ALL (10, 25), raising the possibility of non-genetic inactivation of E2A or HEB that has so far escaped genomic studies. E protein activity can be inhibited by direct heterodimerization with Id proteins, members of the HLH family that lack DNA binding domains (37) or by the SCL and LYL1 oncoproteins (39, 40, 46, 47). Nonetheless, inhibition of E protein by SCL is insufficient for T-cell leukemogenesis which requires transcription activation of a stemness gene expression program by the SCL-LMO1 complex (2) or LMO2 (48, 49). Finally, O’Neil et al. have previously shown a genetic collaboration between *Tcf12* or *Tcf3*-deficiency and *SCL/TAL1* in accelerating T-ALL onset (47). Because *Heb* deficiency would cause reduced pre-TCR expression (50) and decreased cell proliferation (51), it remains to be documented how this would accelerate T-ALL onset. In summary, while it is well recognized that E2A can be a tumor suppressor in mouse models, it is not clear whether E2A or HEB is inactivated in human T-ALL and how inactivation may occur, given the essential and dosage-dependent role of E2A and HEB in the T lineage (50–52).

Given the intricate interaction between NOTCH1, pre-TCR signaling and E proteins, we elected to use *Cd3e*^-/-^ mice as a powerful genetic model to dissect and decouple the roles of NOTCH1, pre-TCR and HEB in leukemia progression, specifically in DN3 thymocytes, previously shown to be the cell of origin of SCL-LMO1 (2, 4) and LMO2 (48) -induced T-ALL. Thus, by abrogating β-selection and analyzing *Notch1* gain of function and *Tcf12* loss of function individually, our results unravel a strong selective pressure for down regulation of HEB protein levels driven either by NOTCH1 and/or by pre-TCR signaling as a requirement for progression from the pre-leukemic state to overt T-ALL.

## 2 Materials and Methods

### 2.1 Mouse Models and Cell Lines

All animals were kept on a C57BL6/J strain background and maintained in pathogen-free conditions according to institutional animal care and use guidelines. Lck-NotchIC9 (*Notch1*^tg^) (53), SIL-SCL (A (5)3SCL; *SCL*^tg^) (54), *Lck-LMO1* (*LMO1*^tg^) (55), *Cd3e*^-/-^ (56), *E2a*/*Tcf3*^+/-^ (57) and *Heb/Tcf12*^+/-^ (52) were described previously. Kaplan-Meier survival and statistical analysis was performed using GraphPad Prism 9.0 software (GraphPad Software, Inc.). T-ALL susceptibility was computed from areas under the curve (AUC) of Kaplan–Meier survival curves using Prism (1-AUC). Generation of human xenograft T-ALL blasts (14H025 and 14H148) were described previously (58). Human blast and primary murine thymocytes were cultured in MEM Alpha culture medium (Thermo Fisher) supplemented with 10% FBS, 10mM HEPES, 1mM sodium pyruvate, 55 μM β-mercaptoethanol, 2 mM glutamax, 5 ng/mL human FLT-3 Ligand, 5 ng/mL murine IL-7 and 20 ng/mL murine SCF. The DN T-cell line AD10.1 and Jurkat were cultured as previously described (50). KOPT-K1 and P12-ICHIKAWA cell lines were obtained from the DSMZ collection, Germany and maintained in RPMI-1640 culture media supplemented with 10% Fetal Bovine Serum (FBS).

### 2.2 Flow Cytometry Analysis and Cell Sorting

Single-cell suspensions were prepared from thymi or thymoma of mice. Flow cytometry analysis and cell sorting were done as described previously (39) using antibodies against Thy1.2, CD4, CD8, CD25 and CD44, using propidium iodide to exclude dead cells. Multiparametric flow cytometry analysis was performed on a FORTESSA flow cytometer, and cell sorting was performed on FACSAria (BD Biosciences, San Jose, CA). Cell cycle analysis using DAPI staining was performed using ModFit (Verity Software Software, USA).

### 2.3 RT-PCR and *Notch1* Sequencing

For *Cdnk1*a gene expression analysis, DN thymocytes from wt and *Heb/Tcf12*^-/-^ newborn mice were sorted by flow cytometry and cDNAs were prepared as described previously (39). Southern blots of the amplicons were revealed by hybridization using an internal ^32^P-labeled oligonucleotide fragment (primer sequences are listed in **Supplementary Table l**). The ribosomal *Rps16* expression was used as a control for cDNA quality and quantity.

For *Notch1* sequencing, cDNA was prepared from total RNAs as described previously (39). Amplification of *Notch1* exons 26, 27, and 34 from leukemias cDNA were Sanger sequenced in both directions. Quantitative gene expression analysis was performed on StepOne system (Life Technologies) using specific primers and Advanced qPCR mix (Wisent). Primer sequences used for specific mRNA amplification are listed in **Supplemental Table S1**.

### 2.4 Western Blot Analysis

Cells were lysed in RIPA buffer containing a cocktail of protease inhibitors. Protein extracts were resolved on bis-acrylamide gel, transferred on PVDF membranes and hybridized with anti-HEB and anti-E2A (Santa-Cruz Biotechnology Inc., CA) and anti-tubulin-β (Sigma) and anti-ERK (Cell Signaling) as a loading control.

### 2.5 ChIP Assays

Chromatin immunoprecipitation were performed on either *Cd3e*^-/-^ primary thymocytes or AD10.1 extracts as previously described (50). Quantitative PCR was performed on StepOne system (Life Technologies) using specific primers using Advanced qPCR mix (Wisent). Oligonucleotide sequences used for promoter amplification are shown in **Supplementary Table 1**.

### 2.6 Microarray Analysis

Total RNAs were prepared from freshly isolated thymocytes from *Cd3e^-/-^* (control DN3 thymocytes), *Cd3^-/-^SCL*^tg^*LMO1^tg^* T-ALL and pre-leukemic (3-week-old), and *Cd3e^+/+^SCL*^tg^*LMO1^tg^* leukemic mice using the RNeasy extraction kit (Qiagen, Mississauga, ON). cDNA synthesis, labeling and hybridization onto Affymetrix Mouse Genome 430A 2.0 arrays were performed at the Ottawa Health Research Institute (Ottawa, ON) as described (2). Raw data were normalized using the RMA procedure implemented in the Affy package from Bioconductor (59).

### 2.7 Gene Set Enrichment Analysis

We obtained raw microarray data for normal thymocyte populations generated by the Immgen project from GEO (accession number GSE15907). Data were normalized using the RMA procedure implemented in the Affy package from Bioconductor (59). We derived “transition” signatures for each differentiation step (i.e. ETP to DN2, DN2 to DN3a, etc.), which contained genes whose expression levels present with at least a 2-fold change (up or down-regulated) in the transition (gene signatures provided in **Supplementary File 1**). In **Figure 1F**, gene set enrichment analysis (60) was applied to detect transition signatures that are enriched in the transcriptome of *Cd3e^-/-^SCL*^tg^*LMO1^tg^* and *Cd3e^+/+^SCL*^tg^*LMO1^tg^* leukemic cells.

Pre-TCR specific signatures (**Figure 1G**, **Supplementary File 1**) included genes that increased or decreased at least 2-fold during the DN3a-DN3b transition, and are not regulated by the αβTCR in peripheral CD8^+^ T cells stimulated by antigen (naive versus activated CD8+ T cells). Conversely, TCR-specific signatures (**Figure 1G**, **Supplementary File 1**) included genes exclusively regulated by the αβTCR in activated CD8^+^ T cells.

### 2.8 Regulator Analysis Using ChIP-Seq Datasets

**Figure 2:**
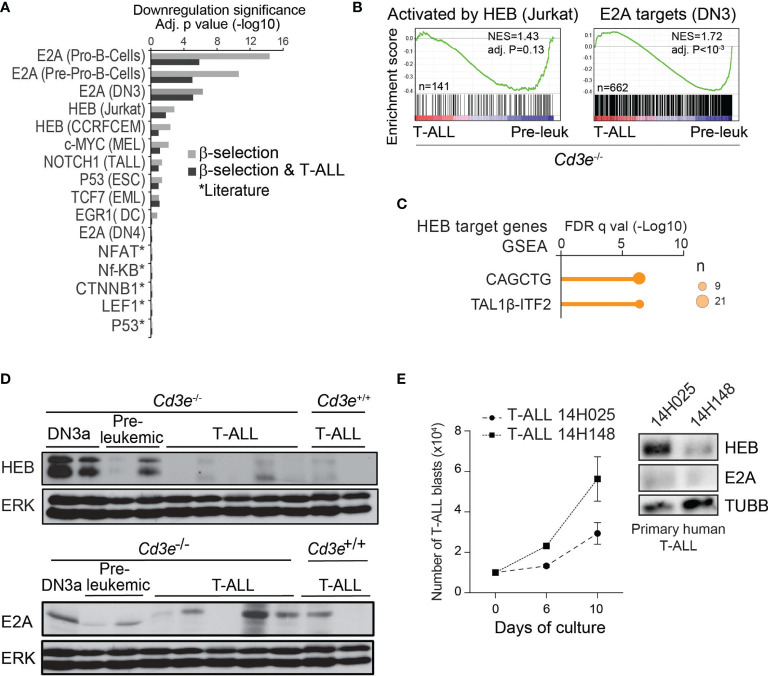
T-ALL progression associated with an inhibition of E proteins targets and downregulation of HEB protein. **(A)** Regulator analysis identified transcription factors associated to transcriptional repression during T-ALL progression and β-selection. Regulators are rank-ordered according to their enrichment scores. The bars display the downregulation significance (log10 adjusted P, Fisher’s exact test). Targets were extracted from ChIP-seq datasets (cell types in parenthesis). **(B)** GSEA analysis of genes activated by HEB and E2A targets during progression from pre-leukemic to leukemic state in *Cd3e*-deficient *SCL*^tg^*LMO1^tg^* T-ALL. **(C)** Binding site motif enrichment (MSig) within the proximal promoter regions of HEB target genes down-regulated between *Cd3e*^-/-^ leukemic and pre-leukemic cells. **(D)** HEB and E2A protein levels during T-ALL progression measured by Western blotting. **(E)** Growth in vitro for primary T‐ALL samples (left panel). Western blot analysis of theindicated T-ALL samples (right panel).

We collected genome-wide chromatin occupancy data for 7 transcription factors implicated in pre-TCR signalling (11 ChIP-seq experiments in total, **Figure 2A**) from Wang et al. (61), Miyazaki et al. (37), and the HemoChIP project (62). ChIP-seq data obtained for E2A (DN3 and DN4) (37) and NOTCH1 (G4A2 and T6E murine cell lines) (61) were processed according to the following steps: (i) sequence reads were mapped to the mouse genome mm9 using Bowtie with default parameters (maximum 2 mismatches); and (ii) peak coordinates were determined by the MACS tool, using the cutoff P < 10^–9^. Peak coordinates for the HemoChIP dataset mapped to the mouse genome mm9 were downloaded from http://hscl.cimr.cam.ac.uk/ChIP-Seq_Compendium/ChIP- Seq_Compendium2.html. Last, all peaks were associated to their closest transcription start sites in the mouse genome using PeakAnalyzer v.1.4 tool (63). Lists of targets bound by transcription factors included all genes containing at least one binding site for the regulator (**Supplementary File 1**). We tested enrichment of targets using the Fisher’s exact test (**Figure 2A**).

### 2.9 Exome Sequencing and Data Analysis

DNA was extracted from *Cd3e^-/-^SCL*^tg^*LMO1^tg^* leukemias (n=4) and control *Cd3e^-/-^* thymocytes (n=2), followed by targeted exome enrichment was performed using the mouse Nimblegen SeqCap EZ kit from Roche. Sequencing was performed in the Illumina HiSeq2000 at the IRIC Genomics platform. Low quality bases (quality below 20) in paired-end reads were trimmed off using the Trimmomatic tool (64), duplicate reads were removed using Picard (http://broadinstitute.github.io/picard), and alignment to the mouse genome (mm10) was performed with bwa (65). The resulting depth of coverage was 20X for at least 85% of the captured exome across all samples. Exome variants in T-ALL samples were predicted using Strelka (66), using *Cd3e^-/-^* thymocytes as matching controls, and annotation was performed using ANNOVAR (67). SIFT scores to determine deleterious variants were computed using the Variant Effect Predictor tool (68).

### 2.10 RNA-Sequencing and Data Analysis

RNA extracted from *Cd3e^-/-^SCL*^tg^*LMO1^tg^* leukemias (n=4) and control *Cd3e^-/-^* thymocytes (n=2) was prepared using the TruSeq RNA kit (Illumina) and sequenced in the Illumina HiSeq2000 in the IRIC Genomics platform. Low quality bases (quality below 20) in paired-end reads were trimmed off using the Trimmomatic, and processed reads were aligned to the mouse genome mm10 using Tophat2/Bowtie2 v2.0.7 (69). Gene levels were quantified (FPKM values) based on the UCSC reference genes annotation using cuffdiff v.2.1.1.

RNA-seq data from pediatric tumors including a minimum of 313 T-ALL, 720 B-ALL and 350 Brain tumor samples were accessible for data analysis *via* the St. Jude PeCan data portal (https://pecan.stjude.cloud) (70) using Protein paint to capture RNA expression from the above ALL datasets (71).

## 3 Results

### 3.1 Functional Importance of the Pre-TCR in Disease Progression

The first critical event in leukemogenesis is the reprogramming of DN3 thymocytes into pre-leukemic stem cells (pre-LSCs) by the SCL-LMO1 oncogenes (2). These two oncogenes target the DN3 population (4, 72) but are not sufficient *per se* for progression to T-ALL. While the initiating reprogramming event is pre-TCR independent (2), progression to T-ALL requires both pre-TCR and NOTCH1 signaling (4), thus emulating the requirement for cooperative signaling between the two important pathways for normal thymocyte differentiation (11). To define the precise contribution of each pathway to disease progression, we took a genetic approach to quantitatively estimate T-ALL progression using disease penetrance and the time to leukemia onset as endpoints to measure leukemogenesis. T-ALL induced by *SCL*^tg^*LMO1*^tg^ or *Notch1*^tg^ separately is affected by the absence of pre-TCR/CD3 signaling (**Figure 1B**) as previously reported (4, 72, 73). In contrast, *SCL-LMO1* together with the hyperactive *Notch1* allele (*ICN1*, hereafter *Notch1*^tg^), induce T-ALL with full penetrance in the presence or in the absence of *Cd3e* (**Figure 1C**). Nonetheless, in *Cd3e*-proficient mice, pre-TCR/CD3 signaling accelerates the disease to 29 days, compared to 112 days in *Cd3e*-deficient mice. These results indicate that *Notch1*^tg^ drives the penetrance of T-ALL while the pre-TCR determines the time to leukemia. Of note, T-ALL induced by the three oncogenes together in the absence of *Cd3e* reproduce the disease induced by the two transcription factor oncogenes *SCL* and *LMO1* in a *Cd3e*-proficient background (**Figure S1**). Therefore, in the context of T-ALL induced by the *SCL* and *LMO1* oncogenes, the *Notch1* transgene controls disease penetrance while pre-TCR signaling accelerates disease onset.

### 3.2 Re-Activation of a Pre-TCR-Driven Proliferation Signature in the Absence of CD3 Signaling Associated With Disease Progression

While pre-TCR signaling has been known to be important for leukemogenesis (74, 75) and more specifically for SCL-LMO1-induced T-ALL (4, 76), the contribution of the pre-TCR and downstream molecular effectors remain to be uncovered. T-ALL that still develops in the absence of CD3 or RAG, completely lacked the typical *Notch1* gain of function mutations (4), providing us with a unique genetic tool to dissect the contribution of these pathways to T-ALL.

We therefore conducted a transcriptomic analysis that capitalized on our identification of DN3a as the cell of origin of T-ALL, and in *Cd3e*^-/-^ mice in which pre-TCR signaling is abrogated, causing thymocyte differentiation blockade at the DN3a stage. This allows for a stringent comparison between the pre-leukemic and the leukemic state to define the molecular signature of progression. During the pre-leukemic stage, *Cd3e^-/-^SCL*^tg^*LMO1^tg^* thymocytes are blocked at the DN3a stage, as expected from the absence of CD3.

We next compared the transcriptomes of pre-leukemic (n=3) and of *SCL^tg^LMO1*^tg^ leukemic cells (n=6) (**Figure 1D**). We applied principal component analysis (PCA) to compare the transcriptomes of normal thymocyte populations (obtained from the Immgen project) with pre-leukemic and leukemic samples. The first component reflected the distinct Affymetrix chips used for profiling whereas the second PCA component organized the transcriptomes according to their differentiation trajectories, from ETP to DP cells. Overall, PCA showed that pre-leukemic samples were comparable to the *Cd3ϵ*^-/-^ DN3a thymocytes. Strikingly, progression to T-ALL correlates with the expression profiles of thymocytes that have undergone β-selection, despite the absence of pre-TCR signaling in *Cd3ϵ*^-/-^ mice. Consistent with PCA, we observed that leukemic cells acquired a post-β-selection phenotype to become DN3b-DP cells (98% of the thymic mass), compared to 2% at the pre-leukemic stage, despite the complete lack of normal pre-TCR function in *Cd3e*^-/-^ mice (**Figure 1E**).

We also correlated disease progression with gene signatures of thymocyte differentiation using gene set enrichment analysis (GSEA). We observed that only signatures associated to post-β-selection thymocytes (DN3b to DN4 cells) were positively correlated with T-ALL progression (**Figure 1F**). The strongest correlation was associated with the DN3a-DN3b transition, exactly at the stage where the pre-TCR/CD3 triggers a burst of cell proliferation following a productive TCRβ rearrangement and the formation of a functional pre-TCR/CD3 complex.

CD3 signaling is important for pre-TCR function and β-selection, but also for TCR signaling. To distinguish the contribution of these two pathways to T-ALL progression, we applied GSEA to analyse gene signatures of antigen-independent (pre-TCR) and antigen-dependent (TCR) T-cell stimulation in T-ALL progression. Pre-TCR-induced genes (DN3b-DN4) correlated positively with leukemia progression (**Figure 1G**, adj. P < 10^-4^). In contrast, the TCR gene signature did not correlate positively with T-ALL progression induced by SCL-LMO1 (**Figure 1G**).

In summary, our results indicate that leukemic cells display gene signatures of post-β-selection thymocytes, suggesting that a pre-TCR/CD3-like proliferation has occurred even in the absence of a functional pre-TCR (*Cd3e*^-/-^ background). Moreover, progression to T-ALL overlaps specifically with pre-TCR-driven gene signature. This CD3-independent activation of the pre-TCR molecular signature indicates a strong selective pressure during leukemic progression for pathways that normally control the β-selection checkpoint.

### 3.3 T-ALL Progression Associated With an Inhibition of E Proteins

#### 3.3.1 Genomic Analyses Identify the Down-Regulation of E2A and HEB Targets During Normal β-Selection and the Progression From Pre-Leukemic to Leukemic Stages

Several transcription factors have been implicated in pre-TCR signaling and/or β-selection (reviewed in (77)). To determine their potential contribution to T-ALL progression, we first performed a systematic regulator analysis based on published ChIP-seq datasets (62).

This analysis predicted that targets of E proteins, E2A and HEB, are down-regulated during β-selection and T-ALL progression (**Figure 2A**, Fisher’s exact test). In addition, from data obtained with shRNA knock-down of *HEB* (78),we identified a list of 389 genes activated by HEB in Jurkat cells, (i.e. fold-change > 1.5, t-test P < 0.05). GSEA indicated that these HEB targets (**Figure 2B**, left panel) as well as E2A-bound genes (**Figure 2B**, right panel) are down-regulated when comparing *Cd3e*^-/-^ leukemic and pre-leukemic cells. Last, well known E-Box motifs CAGCTG and TAL1β-ITF2 (79) were found to be enriched in HEB target genes (**Figure 2C**).

Overall, these analyses indicate that inhibition of E protein activity may be important both at the β-selection checkpoint and during T-ALL progression.

#### 3.3.2 HEB Protein Levels Are Down Regulated in T-ALL

Mice lacking *E2a*/*Tcf3* develop lymphomas, suggesting that E2A is a tumor suppressor (42, 45). Nonetheless, *TCF3* mRNA is highly expressed in human T-ALL (9, 80) and the *TCF3* gene is neither deleted nor mutated, raising the question how E2A acts as a tumor suppressor. Previous work showed that pre-TCR signaling inhibits E2A activity *via* upregulation of *Id3* (81). However, the very low levels of *ID3* in most human T-ALL samples (**Figure S2A**) and in murine *SCL^tg^LMO1^tg^* T-ALL (**Figure S2B**) do not support a role for ID3 in sequestering HEB or E2A in T-ALL.

Next, we investigated *Heb* expression at the mRNA and protein levels in T-ALL progression. *Heb/Tcf12* mRNA levels were equally high in control, pre-leukemic and leukemic cells (**Figure S2C**). In contrast, we found by western blotting that HEB protein was almost absent in murine leukemic cells, contrasting with high expression levels in normal DN3a thymocytes and variable levels in pre-leukemic thymocytes (**Figure 2D**, upper panel). E2A protein levels also decreased with progression to T-ALL, albeit to a lesser extent (**Figure 2D**, lower panel) while mRNA levels remained elevated (**Figure S2C**). Moreover, we observed that HEB levels steadily increased in Jurkat cells treated with the proteasome inhibitor MG132 (**Figure S2D**), indicating that HEB levels are regulated by proteasomal degradation in leukemic cells. We next inspected E protein levels in two primary T-ALL patient samples. HEB protein levels were undetected in the sample with higher proliferation in culture, whereas E2A was undetectable in both samples (**Figure 2E**). Taken together, these results indicate a strong selective pressure for HEB protein down-regulation during progression to T-ALL.

### 3.4 HEB Restricts Cell Proliferation at the β-Selection Checkpoint and Acts a Tumor Suppressor in T-ALL

**Figure 3:**
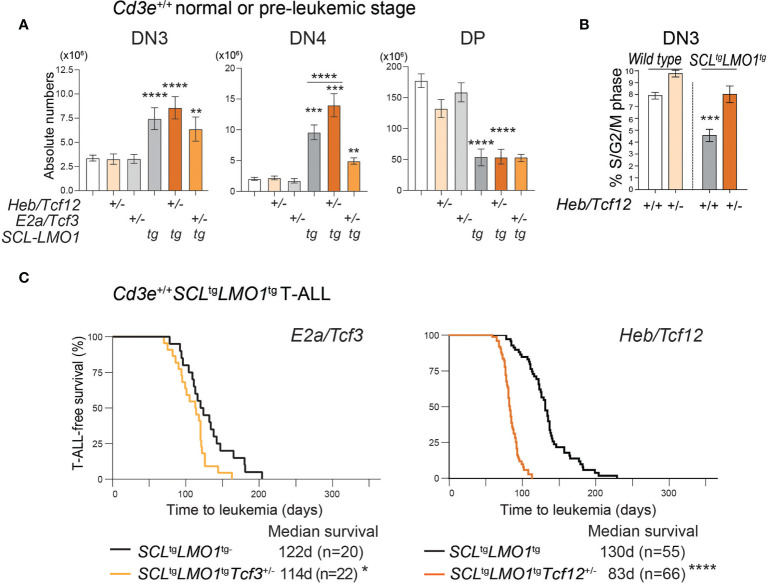
HEB restricts cell proliferation at the b‐selection checkpoint and acts as a tumor suppressor in T-ALL. **(A)** Effect of loss of one allele of *Heb*/*Tcf12* or *E2a*/*Tcf3* on the absolute numbers of thymocytes within the indicated subsets in a *wt* or *SCL*^tg^*LMO1^tg^ background.* Shown are the average ± SD of at least 9 mice per group (5-6 weeks). Where not specified P value is compared to wild type control: **p = 0.0039, ***p < 0.002 and ****p < 0.0001. **(B)** Monoallelic Heb/Tcf12 deletion increased S/G2/M phase in Cd3e-deficientpre-leukemic DN3 thymocytes. Cell cycle analysis of Cd3e-deficient thymocytes in Heb/ Tcf12+/+ andHeb/Tcf12+/- backgrounds. *p value <0.01). **(C)** Kaplan-Meyer survival curves comparing *E2a/Tcf3*^+/+^ with *E2a/Tcf3*^+/-^ backgrounds (left panel) as well as *Heb/Tcf12^+/+^* and *Heb/Tcf12^+/-^* backgrounds in Cd3*e*-proficient *SCL*^tg^*LMO1^tg^* leukemias. In both panels, the +/+ genotypes are wild type littermates of +/- mice. N represents the numbers of mice and the median survival in days was computed from the survival curves. *p = 0.039 and ****p < 0.0001.

To directly address the role of *Heb* or *E2a*, we analyzed thymocyte numbers in *E2a/Tcf3*^+/-^ or *Heb/Tcf12*^+/-^ mice in the context of the *SCL*^tg^*LMO1*^tg^ mice or their wild type (*wt*) littermates. In adult *wt* mice, removal of one *Heb* allele or one *E2a* allele did not significantly affect thymocyte numbers at the DN3 to DP stages (**Figure 3A**), although *Heb* monoallelic deletion resulted in modest but significantly increased cell numbers within populations undergoing β-selection, i.e. DN3b and DN3-4 (**Figure S3**), concurring with the view that *Heb* enforces a proliferation checkpoint at this stage (44, 82). We and others previously showed that the *SCL-LMO1* or *LMO2* oncogenes expand the DN3 populations due to increased self-renewal capacity (2, 4). Interestingly, *Heb* haploinsufficiency further increased the expansion of the DN3 and DN4 populations induced by the *SCL-LMO1* oncogenes (**Figure 3A**). To directly address the antiproliferative role of HEB, we compared S/G2/M phase progression in *Heb/Tcf12*^+/-^ and *Heb/Tcf12*^+/+^ DN3 thymocytes from *Cd3e*^-/-^ mice. In absence of pre-TCR signaling, while loss of one allele of *Heb* increased the proportion of proliferating cells, we found that the *SCL-LMO1* oncogenes decreased the proportion of cycling DN3 thymocytes, consistent with a role for *SCL* in quiescence control (83). In this context, removing one *Heb* allele re-established proliferating DN3 thymocytes to normal proportions (**Figure 3B**). Therefore, in the absence of pre-TCR signaling, the SCL and LMO1 oncogenes revealed *Heb* haplo-insufficiency in cell cycle control, indicating and that HEB anti-proliferative function is required to control oncogenic stress at the β-selection checkpoint. In addition to the previously reported role for *Id3*-mediated inhibition of E proteins during β-selection in steady state (84), our data indicate a distinctive requirement for HEB in stress response.

The E proteins and Id axis has a well-established tumor-suppressor function (42, 45). We therefore addressed the question whether both HEB and E2A have a tumor suppressor function in the context of SCL-LMO1-induced T-ALL (**Figure 3C**). In *Cd3e*-proficient mice, loss of one *E2a* allele caused a modest decrease in latency from 122 days in littermate controls to 114 days (**Figure 3C**, left panel). In contrast, deletion of one *Heb* allele accelerated the time of onset to 83 days compared to 130 days in littermate controls (**Figure 3C**, right panel). Together, our results indicate a tumor suppressor function for *Heb* which acts in a gene-dosage dependent manner in T-ALL induced by SCL and LMO1.

### 3.5 Monoallelic *Heb/Tcf12* Deletion Accelerates *SCL*^tg^*LMO1*^tg^-Induced T-ALL Without Affecting *Notch1*^tg^-Induced T-ALL

**Figure 4:**
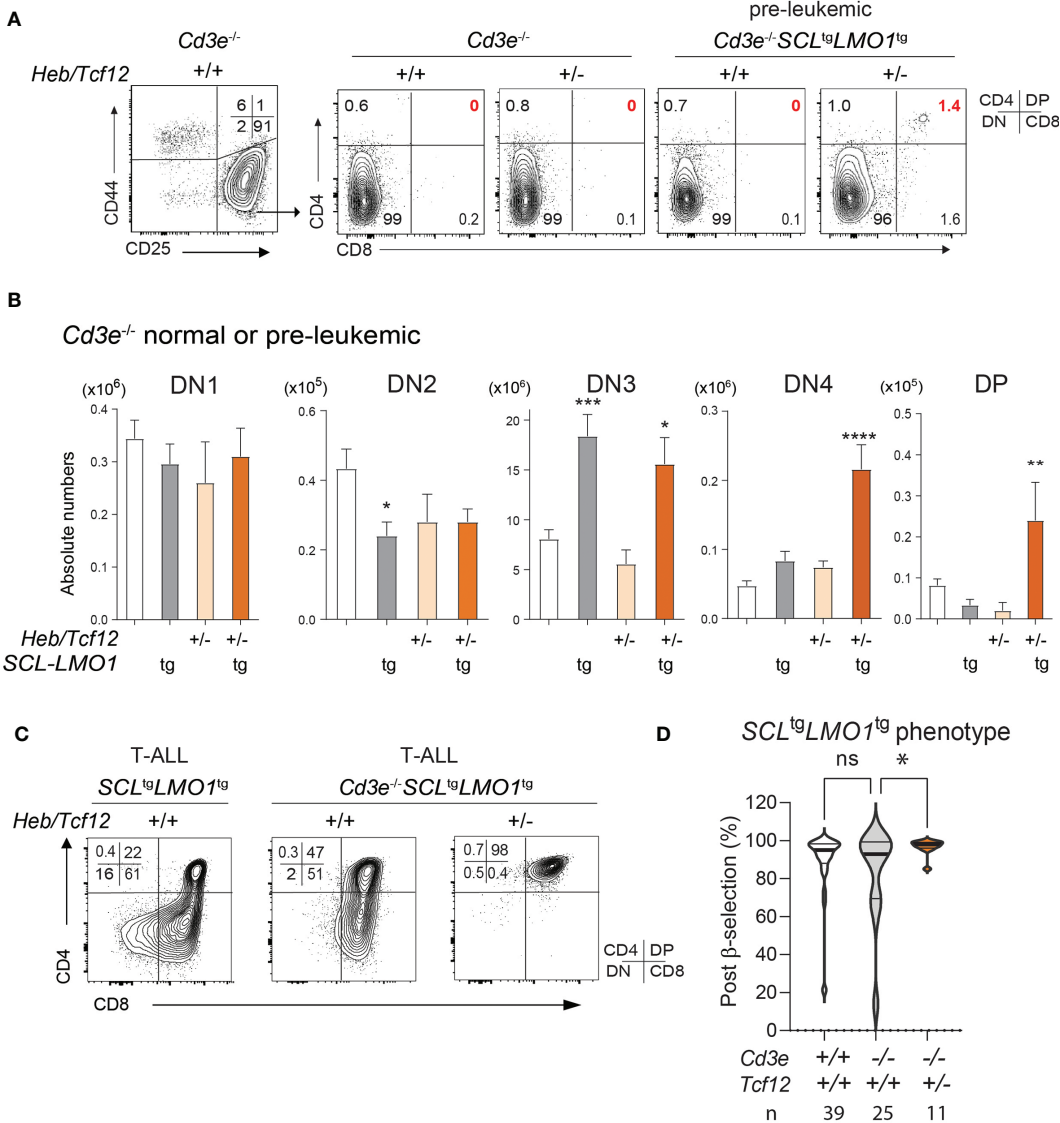
The SCL and LMO1 oncogenes favor the DN-DP transition at the leukemic stage despite the absence of pre-TCR/CD3 signaling: synergy with decreased *Heb* gene dosage. **(A)** FACS phenotypes of normal *Cd3e^-/-^* or pre-leukemic *Cd3e^-/-^SCL*^tg^*LMO1^tg^* thymocytes from *Heb/Tcf12^+/+^* and *Heb/Tcf12^+/-^* backgrounds. Flow cytometry profiles for the CD4− CD8− DN populations are shown in **Figure S4**. **(B)** Absolute numbers of thymocytes within the indicated subsets in normal *Cd3e^-/-^* or pre-leukemic *Cd3e^-/-^SCL*^tg^*LMO1^tg^* in *Heb/Tcf12^+/+^* and *Heb/Tcf12^+/-^* backgrounds. Shown are the average ± SD of at least 5 mice per group, taken at 4 weeks. Note the significant increase in the post-β selection DN4 and DP populations in *SCL*^tg^*LMO1*^tg^*Heb/Tcf12*^+/-^ mice. **(C)** FACS phenotypes of *Cd3e*-proficient or deficient *SCL*^tg^*LMO1^tg^* leukemias from *Heb/Tcf12^+/+^* and *Heb/Tcf12^+/-^* backgrounds. **(D)** T-ALL with a post-β-selection phenotype. Shown are the percentages of cells from each T-ALL with post-β-selection surface phenotypes (DN3-4, DN4, ISP, SP). n represents the numbers of mice analysed. * adj p=0.02. pvalue < 0.05; **p value < 0.005; ***p value < 0.0005; ****p value < 0.0001, ns, Non significant.

In *Cd3e*-deficient mice expressing the *SCL* and *LMO1* oncogenes, inactivation of a single *Heb* allele bypassed pre-TCR signalling to increase the proportion and numbers of DN4 cells during the pre-leukemic stage (**Figures 4A, B** and **Figure S4**). In addition, monoallelic *Heb* deletion allowed a minor population of SCL-LMO1 expressing thymocytes to progress to the DP stage (1.4%, **Figures 4A, B**). Last, progression to the leukemic stage is associated with a transition to a post-β-selection phenotype in *Cd3e*-deficient *SCL*^tg^*LMO1*^tg^ mice, which consistently increased in *Heb/Tcf12*^+/-^ T-ALL (**Figures 4C, D**).

**Figure 5:**
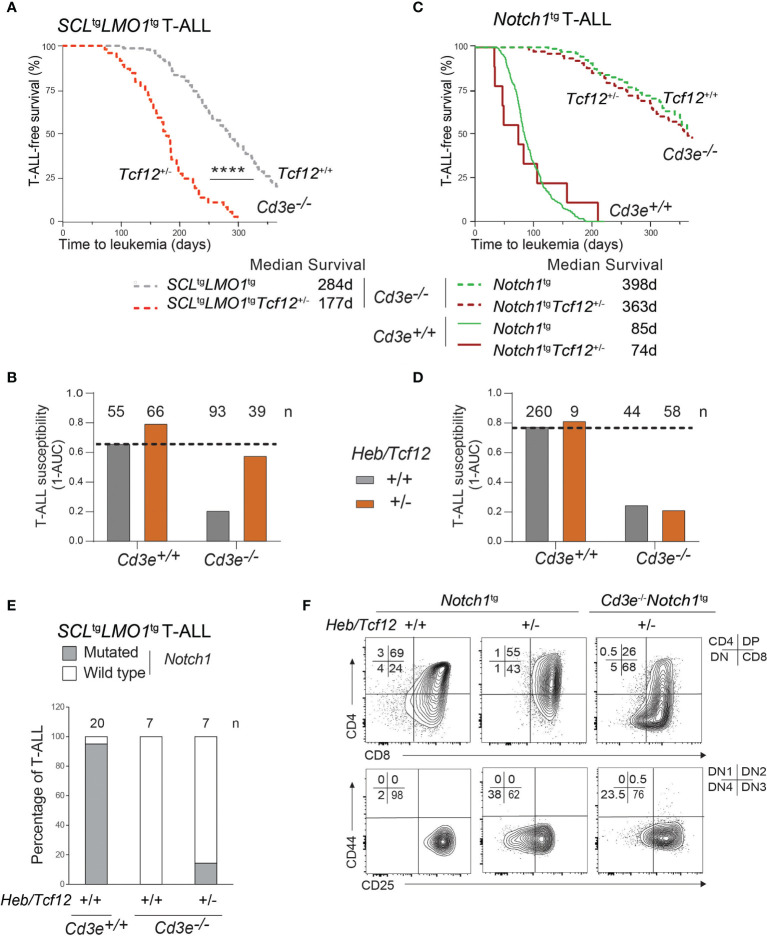
The *SCL* and *LMO1* oncogenes but not the *Notch1/IC9* oncogene reveal *Heb/Tcf12* as a haplo-insufficient tumor suppressor in T-ALL. **(A)** Kaplan-Meyer survival curves comparing *Cd3e*-deficient *SCL*^tg^*LMO1^tg^* leukemias in *Heb/Tcf12^+/+^* and *Heb/Tcf12^+/-^* backgrounds. Shown is the median survival in days. **(B)** T-ALL susceptibility of *SCL*^tg^*LMO1*^tg^ mice in *Cd3e*-proficient (*Cd3e*^+/+^, **Figure 3C**) or deficient (*Cd3e*^-/-^, **Figure 5A**) backgrounds was calculated from the area under the curve (AUC) of the above Kaplan-Meyer graph over 365 days. Shown on top are the numbers of mice per group. **(C, D)** Kaplan-Meyer survival curves **(C)** and T-ALL susceptibility **(D)** comparing *Notch1/IC9-*induced leukemias in *Heb/Tcf12^+/+^* and *Heb/Tcf12^+/-^* backgrounds that are *Cd3e*-deficient or *Cd3e*-proficient. **(E)** Presence of *Notch1* activating mutations in *Cd3e*-proficient but not *Cd3e*-deficient *SCL*^tg^*LMO1^tg^* T-ALL. Shown are the percentage of T-ALL samples with an activating mutation in the *Notch1* locus in *Heb/Tcf12^+/+^* and *Heb/Tcf12^+/-^* backgrounds. Shown on top are the numbers of leukemias sequenced in each group. Only one *Heb/Tcf12*^+/-^*Cd3e*-deficient *SCL*^tg^*LMO1^tg^* T-ALL harbours a mutation affecting the PEST domain of *Notch1.* **(F)** Leukemic phenotypes of *Cd3e*-proficient or *Cd3e*-deficient *Notch1^tg^* leukemias from *Heb/Tcf12^+/+^* and *Heb/Tcf12^+/-^* backgrounds **** pvalue<0.0001.

We next assessed the impact of *Heb* gene dosage on disease penetrance and time to leukemia. Strikingly, decreased *Heb* (*Heb/Tcf12*^+/-^) compensates for the absence of pre-TCR signaling in *Cd3e*^-/-^ mice and allowed *SCL^tg^LMO1^tg^* T-ALL to become fully penetrant, in addition to accelerating disease onset by 107 days (**Figures 5A, B**). Hence, in *Cd3e*^-/-^*SCL^tg^LMO1^tg^* mice in which disease penetrance was 80%, loss of one *Heb* allele recapitulated the effect of the *Notch1* oncogene on restoring full disease penetrance as shown in **Figure 1C**. Since *Notch1*^tg^-induced T-ALL is also dependent on pre-TCR function (**Figures 1C** and **5C, D**), we next addressed the importance of *Heb* in T-ALL induced by *Notch1*^tg^ in pre-TCR proficient and pre-TCR deficient mice. In contrast to *SCL* and *LMO1*, decreased *Heb* gene dosage did not affect *Notch1*^tg^-induced T-ALL in *Cd3e*^-/-^ or in *Cd3e*^+/+^ mice (**Figures 5C–F**), indicating that *Heb* and *Notch1* operate in the same genetic pathway in T-ALL, as suggested during normal differentiation (36). In summary, *Heb* and *Notch1* inversely control the penetrance of T-ALL induced by the SCL and LMO1 oncogenes whereas the pre-TCR determines the DN-DP transition during the pre-leukemic stage and the time of disease onset.

Since the *Notch1* oncogene is sufficient to bypass pre-TCR signaling to confer full leukemic penetrance in *Cd3e*^-/-^*SCL*^tg^*LMO1*^tg^ mice (**Figure 1B**), we addressed the question whether the increased penetrance caused by monoallelic *Heb* deletion shown in **Figure 5A** could be due to the acquisition of *Notch1* gain of function mutations. We analyzed a cohort of mice with *Heb/Tcf12*^+/-^ and *Heb/Tcf12*^+/+^ T-ALL for the presence of *Notch1* mutations (**Figures 5E** and **S5**). As expected, 19 of 20 *SCL*^tg^*LMO1*^tg^ T-ALL in *Cd3e*^+/+^*Heb/Tcf12*^+/+^ mice exhibit *Notch1* gain of function mutations (4, 85) which affect the PEST domain whereas *Cd3e*-deficient *SCL*^tg^*LMO1*^tg^*Heb/Tcf12*^+/+^ T-ALL completely lacked *Notch1* mutation as reported (4). In comparison, only 1 of 7 *Heb/Tcf12*^+/-^ T-ALLs acquired *Notch1* mutations (**Figure 5E** and **Figure S5**). Therefore, the increased aggressiveness of *Heb/Tcf12*^+/-^ T-ALL is unlikely due to *Notch1* gain of function mutations.

These results establish *Heb* as a tumor suppressor that normally enforces a proliferative checkpoint during β-selection to suppress oncogene-induced T-ALL in a gene dosage-dependent manner. Unlike classical tumor suppressors, the human *HEB* gene is not affected at the genomic level in T-ALL (10, 25). Here, we show that HEB is regulated at the protein level, pointing to a distinctive loss of function mechanism.

### 3.6 A Threshold-Dependent Role for *Cdkn1a* Downstream of HEB

#### 3.6.1 Exome Sequencing of *Cd3e^-/-^SCL*^tg^*LMO1^tg^* T-ALLs Reveals Loss of Function Mutations in HEB-Bound Genes

Since *HEB/TCF12* is not deleted nor inactivated by deleterious mutations in human T-ALL, we addressed the possibility that down-regulation or genetic inactivation of HEB targets with tumor suppressor function could also be a mechanism associated to and/or selected for during disease progression. We performed exome sequencing of *Cd3e^-/-^SCL*^tg^*LMO1^tg^* leukemias (n=4) and control *Cd3e^-/^* thymocytes to identify genetic alterations involved in leukemia development in the absence of pre-TCR signaling. The software Strelka (66) was applied to discover somatic SNVs and short indels using the paired tumor-control configuration. After filtering out known polymorphisms reported in the SNP database v.138, we obtained 85 non-synonymous SNVs predicted to be deleterious by the Variant Effect Predictor tool (68). In addition, Strelka predicted 19 stop gains, 10 stop loss, 2 frameshift deletions and 1 frameshift insertion (**Supplementary File 2**).

**Figure 6:**
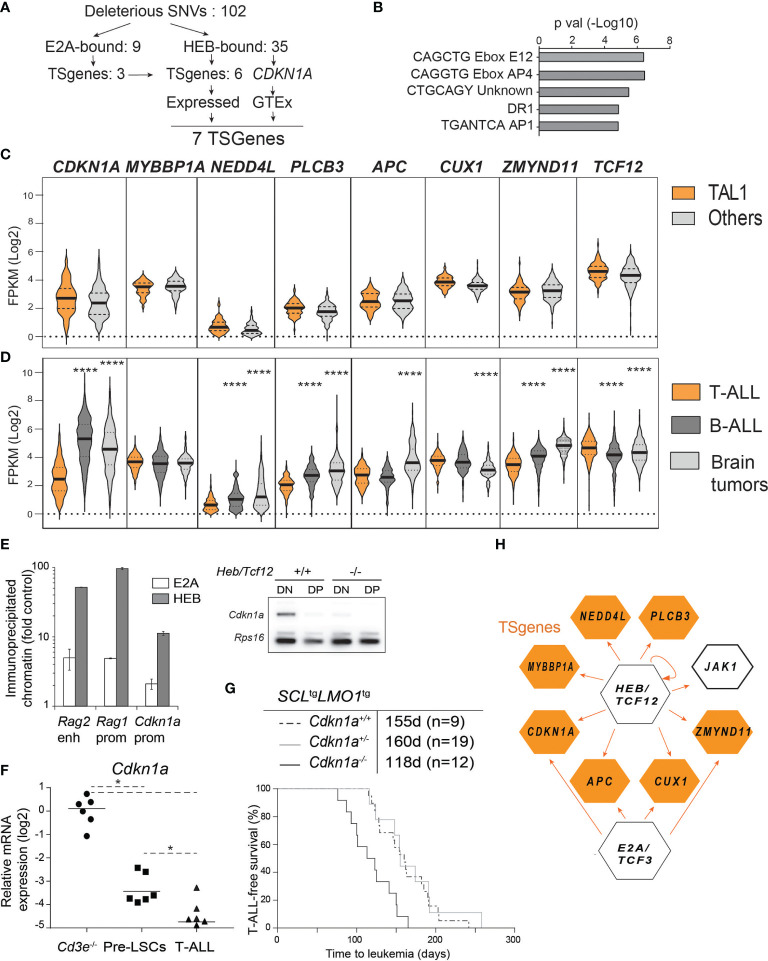
HEB controls a tumor suppressor network in T-ALL. **(A)** Strategy of tumor suppressor identification amongst HEB target genes that are mutated in *Cd3e^-^*^/-^*SCL^tg^LMO1^tg^* leukemic cells. **(B)** Enrichment in E boxes within the proximal promoter regions of HEB target genes. **(C, D)** Expression levels of the indicated TSGenes in TAL1+ vs other molecular subgroups in pediatric T-ALL dataset from Liu et al. **(C)** and in T-ALL, B-ALL and brain tumors from the Pediatric Cancer Genome Project cohort **(D)**. **(E)** Chromatin immunoprecipitation of *Cdkn1a* promoter with HEB and E2A in DN thymocytes. The *Rag1* promoter and *Rag2* enhancer are included as positive controls (left panel) and RT-PCR analysis of *Cdkn1a* expression in DN and DP thymocytes in *wt* and *Heb/Tcf12* knockout mice. Amplified bands were revealed by hybridization with an internal ^32^P-labeled oligonucleotide fragment (right panel). **(F)** Quantitative RT-PCR analysis of *Cdkn1a* expression in *Cd3e^-/-^* DN3 cells, pre-LSCs and leukemic cells. **(G)** Kaplan-Meyer survival curves of *SCL*^tg^*LMO1^tg^* T-ALL with partial and complete loss of *Cdkn1a*. **(H)** A tumor suppressor network downstream of HEB or E2A validated by chromatin immunoprecipitation. HEB is autoregulatory but not E2A. *p value < 0.01; ****p value < 0.0001

We investigated the gene set affected by the above mutations (102 genes in total, **Figure 6A**) using the MSig database enrichment tool (http://software.broadinstitute.org/gsea/msigdb/annotate.jsp). We found a strong enrichment (adjusted P = 1.2x10^-4^) for genes containing the E-box motif CAGGTG in their proximal promoters, suggesting they could be regulated by E proteins E2A and HEB (**Figure 6B** and **Supplementary File 2**). The AP4 E-box motif CAGCTG was also found to be enriched in promoters of mutated genes (35 genes, adjusted P = 1.2x10^-4^).

To confirm binding, we performed ChIP in the AD10 DN cell line and found by PCR that HEB occupies the promoter regions of 29 of the mutated genes within 2 kb of the transcriptional start site, in addition to *Cdkn1a*, validated here as a HEB target (**Figure S6A**). Binding enrichment was at least five-fold over a negative control and for 3 genes, the enrichment was almost as high as that observed with *Ptcra* which was amplified as a positive control (**Figure S6A**). In contrast, *Csf3r* which was amplified as a negative control did not show any enrichment.

#### 3.6.2 Downstream HEB Targets With Putative Tumor Suppressor Function in Human T-ALL

We next addressed whether HEB-bound genes with loss of function mutations in murine T-ALL might be tumor suppressors in the human disease. We included *Cdkn1a* in this analysis because *Cdkn1a* is a well-documented HEB and E2A target gene (41, 44, 86) and because of strong genetic evidence for *Cdkn1a* tumor suppressor function in mice harboring one additional *Cdkn1a* allele (87). Out of 102 genes with deleterious mutations (for a total of 103 including *Cdkn1a*), 35 are HEB-bound genes (34.3%) according to previously published HEB ChIP-seq data with two human TAL1/SCL T-cell lines (78) and ChIP-PCR validation from this study (**Figure S6A**). Of note, 7 of these 35 HEB-bound genes (20%) are known tumor suppressors (https://bioinfo.uth.edu/TSGene, **Figure 6A**). We checked gene expression in blood and brain samples from normal tissues in the GTEx portal (https://www.gtexportal.org) and all seven genes were expressed in normal cells and therefore retained for further analysis. When we applied the same filtering strategy to E2A instead of HEB, only 10 of 103 genes are E2A-bound (9.7%) and 4 of the 10 genes are annotated as TSGenes (**Figures 6A, E, H**). All four genes are present in the seven HEB-bound TSGenes.

These 7 HEB-targets and TS genes were not found to be recurrently mutated in T-ALL (10). Of note, T-ALL can be classified into distinct molecular subgroups based on chromosomal translocations and gene signatures (9, 23, 88). Since our transgenic model is representative of the SCL/TAL1 molecular subgroup, we next addressed the question whether these seven HEB target TSGenes would be expressed at lower levels in the TAL1 subgroup compared to the other T-ALL subgroups, using the dataset published by Liu et al. (10). Gene expression levels were remarkably comparable between TAL1 and non-TAL1 subgroups and were consistently low compared to *TCF12* (**Figure 6B**). We then searched the Pediatric Cancer Genome Project (https://www.stjude.cloud) (89) that covers more diversified cancer types, in order to compare expression levels for these seven genes in T-ALL (10), B-ALL (90) and brain tumors (**Figure 6D**). Strikingly, *CDKN1A* expression is five-and four-fold lower in T-ALL compared to B-ALL and brain tumors, respectively (**Figure 6D**). In addition, four other TSGenes are expressed at two- to eight-fold lower levels in T-ALL compared to brain tumors and/or B-ALL samples: *NEDD4L*, *PLCB3*, *APC* and *ZMYND11* (**Figure 6D**). These expression patterns contrast sharply with those of *LCK* which is highest in T-ALL as expected (**Figure S6C**), *RUNX1* which is equally expressed in T- and B-ALL but not in brain tumors, or JAK1, encoding a non-receptor tyrosine kinase, which is higher in B-ALL compared to the other two groups, and finally *NAXE*, encoding a metabolic enzyme which is expressed in all three types of pediatric cancers with modest but significantly lower levels in B-ALL (**Figure S6B**). Overall, low expression levels of HEB target TSGenes in primary human T-ALL samples concur with decreased HEB function in T-ALL compared to B-ALL and brain tumors, the three most frequent pediatric cancers, and a tumor suppressor function for HEB in pediatric T-ALL. Of note, these potential TSGenes are rarely mutated or deleted in T-ALL. Rather, these TSGenes are expressed at much lower levels in T-ALL compared to the other pediatric tumors.

#### 3.6.3 *Cdkn1a* Deletion Accelerates *SCL-LMO1*-Induced T-ALL

*Cdkn1a*, a typical target of E2A and Id (41, 86, 91, 92) is part of the gene set that is downregulated at the β-selection checkpoint (**Figure S6C**) (93). p21^Cdkn1a^ mediates G1 arrest by inhibiting CDK1 and CDK2 and loss of *CDKN1A* is a predictor of poor outcome in renal cell carcinoma (94). In agreement with our results (**Figurse 6C, D**), *CDKN1A* was found to be very low in human T-ALL (95). We therefore addressed the functional implication of *Cdkn1a* in this mouse model of SCL/TAL1 human T-ALL. We first confirmed that both E2A and HEB occupy the *Cdkn1a* promoter in primary DN thymocytes (**Figure 6E**, left panel). Moreover, *Cdkn1a* expression was nearly abrogated in *Heb/Tcf12*-deficient DN thymocytes (**Figure 6E**, right panel), indicating that HEB is a major transcriptional regulator of *Cdkn1a* at this developmental stage in the thymus. Treatment of thymocytes with phorbol 12-myristate 13-acetate (PMA) leads to activation of protein kinase C (PKC) which phosphorylates RAF and activates the ERK-MAPK pathway, and thus can be used to emulate pre-TCR/TCR signalling (96). We observed that *Cdkn1a* levels were significantly down-regulated in DN3 cells incubated with PMA for 6 hours (**Figure S6D**), consistent with the view that pre-TCR signals down-regulate p21 through the ERK-MAPK pathway (84, 96). In mouse leukemias, *Cdkn1a* expression was decreased in pre-LSCs compared to wild-type DN3a thymocytes and was further reduced in leukemic cells, in both *Cd3e*^+/+^ and *Cd3e*^-/-^ leukemias (**Figure 6F**). Finally, we observed that T-ALL onset was accelerated by 37 days in *Cdkn1a*-deficient mice (**Figure 6G**), compared to the 53 day acceleration found in *Heb/Tcf12*^+/-^ mice, confirming a tumor-suppressor function for *Cdkn1a* (**Figure 6G**). Of note, *Cdkn1a* was haplosufficient in this genetic assay (**Figure 6G**), indicating that a reduction threshold must be attained for leukemic progression to occur. These results concur with the stepwise decrease in *Cdkn1a* in pre-LSCs and in leukemic blasts at time of overt leukemia, indicating that p21 is a threshold-dependent tumor suppressor in T-ALL. In summary, our genetic approach indicates that in the absence of a hyperactive NOTCH1 and of pre-TCR signaling, progression to acute leukemia in *SCL-LMO1*-induced T-ALL involves the downregulation of a tumor suppressor network implicating HEB and p21^CDKN1A^ (**Figure 6H**).

## 4 Discussion

In the present study, we provide genetic evidence for differing requirements of NOTCH1, pre-TCR signaling and HEB in driving progression to T-ALL. By quantifying two disease endpoints, T-ALL penetrance and disease latency, i.e. the median time to overt leukemia, we show that in the context of the *SCL* and *LMO1* oncogenes, the hyperactive *Notch1* oncogene on its own determines disease penetrance even in the absence of pre-TCR signaling. Despite the apparent sufficiency of the three oncogenes, *SCL*, *LMO1* and *Notch1*, an active pre-TCR accelerates the time of onset to 29 days, which is the time required for a single leukemic stem cell to induce T-ALL upon transplantation as shown previously (4). Therefore, SCL and LMO1 acting in synergy with two essential signaling pathways in thymocyte development are sufficient to transform a normal DN3a thymocyte into a fully transformed leukemia initiating cell. Moreover, our data indicate that *Notch1* and *Heb* operate in the same genetic pathway, and mono-allelic deletion of *Heb* can recapitulate the capacity of the *Notch1* oncogene to cause a fully penetrant disease in the absence of pre-TCR signaling.

### 4.1 Pre-TCR Signaling and Lower HEB Levels as Major Drivers of T-ALL Progression

While pre-TCR signaling has been shown to modulate the aggressiveness of T-ALL in several mouse models, the pre-TCR is dispensable for T-ALL induced by *E47*- or *Trp53*-deficiency (97, 98). Hence, the importance of the pre-TCR in T cell transformation induced by oncogenic events remained to be clarified. We propose that the importance of pre-TCR signaling depends on the primary oncogenic transcription factor. Previous transcriptome analyses of pediatric T-ALL identified five molecular subgroups and showed that the TCR signaling pathway is significantly enriched in the TAL1 group while other KEGG pathways related genes were more generally distributed within the other molecular subgroups (9). We now provide evidence that β-selection but not antigen-specific TCR signals are collaborating events. Indeed, the pre-TCR drives cell proliferation, which is required not only for clonal expansion but also for DN-DP differentiation (99) and thymocyte survival (100). A pre-T cell receptor lacking the TCR beta variable domain causes an expansion of the DP population that precedes overt T cell leukemia, suggesting that abnormal pre-TCR function can be oncogenic (101). In addition, the pre-TCR signal is important for *Notch1/ICN1*, *Notch3*- and *TEL-JAK2*-induced leukemias (74, 102, 103), indicating that developmental processes required for normal thymocyte development can be implicated in the pathogenesis of T-ALL (75). In the present study, even though T-ALL can develop in the absence of *Cd3e* and of *Notch1* mutations, our transcriptomic comparison of preleukemic cells with fully transformed leukemic cells indicate a reactivation of β-selection during the progression to T-ALL. The signal strength providing progression to CD4+CD8+ DP T-ALL can either originate from a hyperactive *Notch1* allele or the deletion of a single *Heb* allele. Our data provide compelling evidence for the importance of signal strength of NOTCH1 or HEB in driving the DN to DP transition associated with leukemogenesis.

The pre-TCR complex induces Id expression and consequently inhibition of E protein activity suggesting that the pre-TCR functions upstream of E proteins (81). Nonetheless, *E2a*-deficient mice develop T-cell lymphomas (42, 45), associated with *Notch1* mutations within the PEST domain (104), suggesting that *E2a* is not downstream of *Notch1*. Rather, the latter observations suggest that *Notch1* and *E2a* operate in parallel pathways. Moreover, *Heb* may function parallel to or downstream of the pre-TCR (51). Since NOTCH1 and pre-TCR functions are cooperative during β-selection (11), and both are believed to affect E protein function (81, 105, 106), pre-TCR signaling becomes a confounding factor in assessing the contribution of NOTCH1 and of E proteins in leukemogenesis. Using *Cd3e*^-/-^ mice in which pre-TCR function is abrogated, we now show that oncogenic *Notch1* controls disease penetrance in *SCL-LMO1*-induced T-ALL while the pre-TCR signal governs the time of leukemia onset. Last, our data indicate that *Heb* but not *E2a* operate in the same pathway as *Notch1* since monoallelic deletion of *Heb* does not affect *Notch1*-induced T-ALL but accelerates the disease induced by SCL-LMO1 in *Cd3e*-sufficient mice. Finally, loss of one *Heb* allele in *Cd3e*-deficient mice compensates in part the *Notch1* oncogene to restore full penetrance to T-ALL induced by *SCL* and *LMO1*.

### 4.2 A Matter of Gene Dosage: Inactivation of HEB Targets in T-ALL by Down-Regulation or Deleterious Mutations.

We and others previously showed that the SCL transcription factor heterodimerizes with E2A or HEB, thereby inhibiting target gene expression such as *Cd4* and *Ptcra* and thymocyte differentiation (39, 40, 50). Nonetheless, E47 deficiency promotes the aberrant development of *Rag1* null thymocytes and appearance of DP cells (84), similar to the appearance of a minor population of DP cells reported here in *Cd3e*-deficient thymocytes lacking one *Heb* allele.

E proteins are negatively regulated by ID proteins, another class of bHLH protein. At the β-selection checkpoint, the activation of RAS extracellular-signal-related kinase (ERK)–MAP kinase pathway upregulates ID proteins that bind to E proteins preventing their transcriptional activity (84). E2A inhibits proliferation and differentiation at the β-selection checkpoint in the absence of pre-TCR expression (84), suggesting that inhibition of E2A *via* Id3 would enforce the two distinctive functional outputs of pre-TCR signaling in favoring both cell proliferation and differentiation. However, Id2 and Id3 suppress lymphomagenesis (107), suggesting a necessary balance of E protein/ID levels during thymocyte differentiation. Partial redundancy may explain that both *E2a* and *Heb* are haplosufficient for thymocyte differentiation during steady-state conditions. Nonetheless, the oncogenic stress induced by SCL and LMO1 in thymocytes reveal *Heb* haploinsufficiency at the DN to DP transition, more specifically at the β-selection checkpoint revealing at the same time that *E2a* cannot compensate for *Heb* in stress-response, despite their quasi-redundancy during steady-state conditions.

Our results also bring out the importance of monitoring protein levels in primary tumors, since inactivation of tumor suppressors *via* protein downregulation could represent a distinct mechanism driving T-ALL.

Master regulators of hematopoietic lineages have been implicated in tumor suppressor function, as reported for C/EBPα (108, 109) and SPI1/PU.1 in acute myeloblastic leukemia (AML) (110) or PAX5 loss of function in B-ALL (90, 111). While *E2A/TCF3* is annotated as a tumor suppressor gene (TSGene 2.0) with direct experimental and clinical evidence, *HEB/TCF12* tumor suppressor function has been overlooked, despite the critical role of HEB in the T lineage (reviewed in (4, 112)). Due to a non-redundant function to enforce T lineage (36) and at multiple stages of T cell differentiation, we propose that the essentiality of *HEB* precludes the possibility of identifying loss of function mutations in hematopoietic malignancies. Our study reveals the possibility of temporary loss of function *via* downregulation of HEB protein levels. Moreover, our analysis of the PCGP cohort identifies decreased gene expression levels in T-ALL compared to other pediatric cancers for at least five TSGenes that are HEB targets, regardless of the T-ALL molecular subgroup (9). In addition to well documented genetic alterations of tumor suppressor genes, our study indicates that reduced expression levels of multiple tumor suppressor genes could also contribute to the process of tumorigenesis, as illustrated here for *CDKN1A*.

### 4.3 *HEB* Controls a Tumor Suppressor Network in T-ALL that Includes *CDKN1*A

*Cdkn1a* is a well known E2A and HEB target (44) and tumor suppressor gene (113). More recently, the *Cdkn1a*^SUPER^ mouse is shown to be more resistant to transformation and exhibit a strong cancer protection phenotype, establishing a direct gene dosage-dependent tumor suppressor function for *CDKN1A* (87). Our observations indicate that *Cdkn1a* is down regulated by the *SCL-LMO1* oncogenes during the pre-leukemic stage and further down regulated during progression to T-ALL. In *Cd3e*-deficient T-ALL, our data point to the down-regulation of *Cdkn1a* as part of the selective pressure to activate molecular mechanisms underlying the β-selection process and drive progression to T-ALL. Supporting this hypothesis, *CDKN1A* gene expression levels in the PCGP cohort is on average six- to seven-fold lower in pediatric T-ALL compared to B-ALL, AML or brain tumors. In addition to mRNA downregulation, p21^Cdkn1a^ can also be phosphorylated and degraded *via* ubiquitin-dependent and ubiquitin-independent proteolysis (113). Much like *HEB*, the *CDKN1A* gene is not subject to copy number variations nor deleterious mutations in hematopoietic malignancies. Rather, CDKN1A levels can be downregulated at the mRNA level, as illustrated here, or at the level of protein stability.

HEB and E2A exert an anti-proliferative function in thymocytes prior to *Tcrb* rearrangement, required for the formation of a functional pre-TCR (44), consistent with a tumor suppressor function reported here for HEB. In addition to a well-timed restriction in cell proliferation, it remains possible that tumor suppression involves additional molecular functions secured either by HEB or HEB downstream targets, as reported for TP53 (114). In addition to *CDKN1A*, HEB and E2A co-occupy three TSG loci, *APC*, a haplo-insufficient tumor suppressor, *ZMYND11* a chromatin reader and tumor suppressor in breast cancer (115) and *CUX1*, a homeodomain transcription regulator and haploinsufficient tumor suppressor (116). HEB is also found to occupy three other TSGenes, *MYBPP1A*, potentially involved in nucleolar stress, *NEDD4L*, an E3 ligase with tumor suppressor function (117) and *PLCB3*, encoding phospholipase C beta 3 (phosphatidylinositol-specific), involved in G-protein-linked receptor-mediated signal transduction (**Figure 6H**). These additional targets may account for the more prominent role of HEB as a tumor suppressor in response to the oncogenic stress induced by SCL and LMO1, exactly at the β-selection checkpoint controlled by HEB. Beyond the SCL/TAL1 molecular group, five HEB target-TSGenes are down regulated in T-ALL compared to other pediatric tumors. In T-ALL, we propose that HEB controls a network of tumor suppressor genes to mitigate the oncogenic stress occurring at the β-selection checkpoint. Unlike classical tumor suppressors, these genes are not inactivated by genomic deletion or deleterious mutations but are down-regulated at the mRNA or at the protein levels. This mechanism cannot be detected by assessing DNA copy number variation or whole-genome sequencing, as exemplified by the total absence of *HEB* copy number loss or point mutations in T-ALL. In this context, future large-scale proteomics studies in primary tumors will be able to address whether downregulation of HEB protein is a recurrent event in human T-ALL.

## Data Availability Statement

The datasets presented in this study can be found in online repositories. The names of the repository/repositories and accession number(s) can be found below: http://www.ncbi.nlm.nih.gov/geo, accession ID: GSE198506. RNA-seq data from pediatric tumors used for analysis in this study were obtained from the St. Jude Cloud (https://www.stjude.cloud).

## Ethics Statement

The studies involving human participants were reviewed and approved by Comité d’éthique de la recherche clinique, Université de Montréal. The patients/participants provided their written informed consent to participate in this study.

## Author Contributions

DV, MT, BG and TH conceived and designed the work. DV, MT, BG, SH and AH conducted the studies and/or contributed to the acquisition of data. DV, MT, BG, SH, PG, SL, JZ-P, JH and JPC contributed to the interpretation of the data and/or data analysis. JH, BG and AH contributed to experiments with primary human leukemic cells. DV, MT, and TH wrote the manuscript. All authors contributed to the article and approved the submitted version.

## Funding

This work was supported by grants from the Canadian Cancer Society (TH), the Canadian Institute for Health Research (TH), the FRSQ Cancer Network (JH), the Leukemia Lymphoma Society of Canada (TH), a post-doctoral fellowship from LLSC (SH) and fellowships from the Cole Foundation (DV and MT). The infrastructure was supported in part by an FRSQ group grant and the Canadian Foundation for Innovation.

## Conflict of Interest

The authors declare that the research was conducted in the absence of any commercial or financial relationships that could be construed as a potential conflict of interest.

## Publisher’s Note

All claims expressed in this article are solely those of the authors and do not necessarily represent those of their affiliated organizations, or those of the publisher, the editors and the reviewers. Any product that may be evaluated in this article, or claim that may be made by its manufacturer, is not guaranteed or endorsed by the publisher.
