# Supplementary figures and images for: Monoallelic Heb/Tcf12 Deletion Reduces the Requirement for NOTCH1 Hyperactivation in T-Cell Acute Lymphoblastic Leukemia
Source: Front Immunol. 2022 Mar 24;13:867443. doi: 10.3389/fimmu.2022.867443 (PMC8987207; doi:10.3389/fimmu.2022.867443)

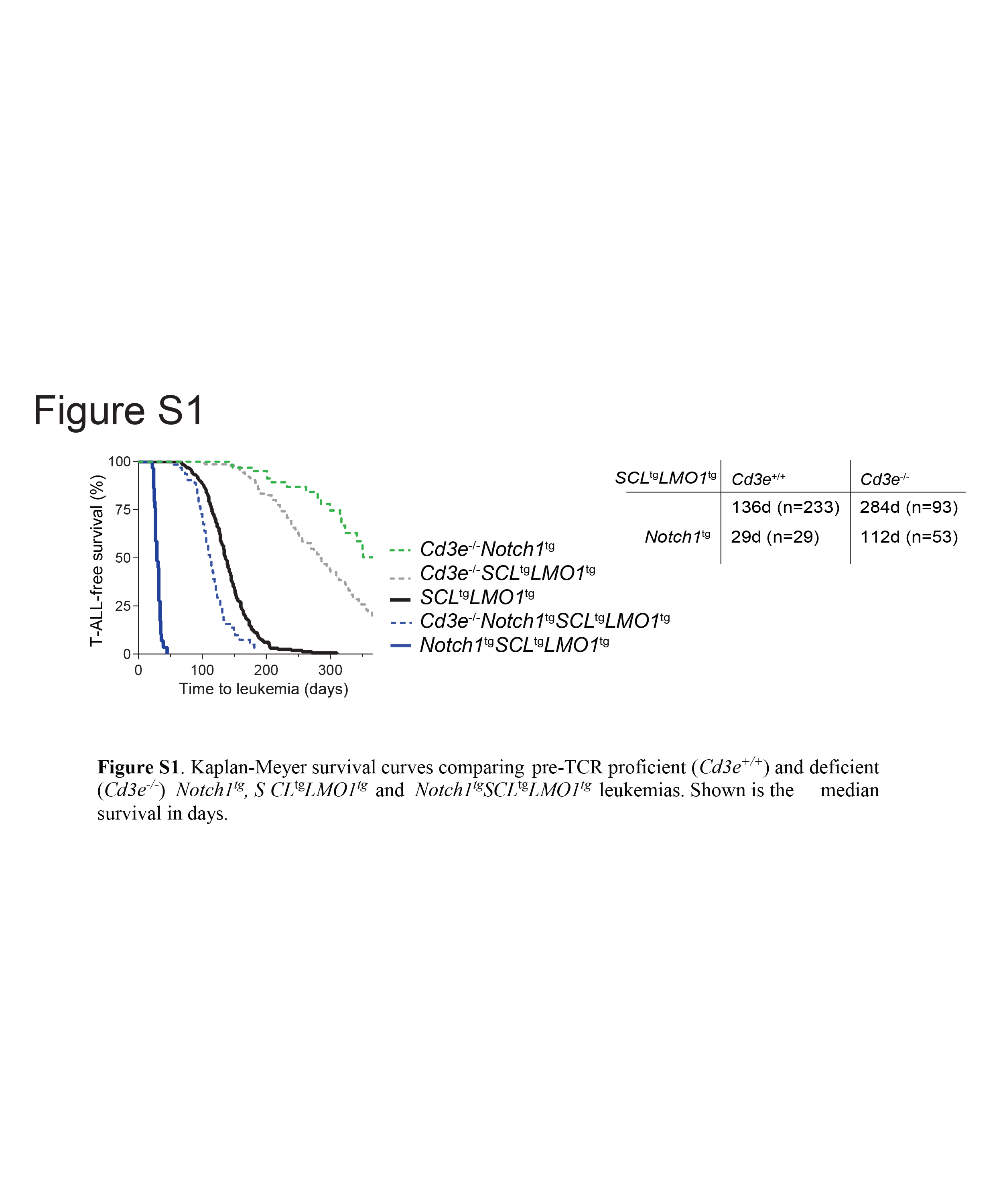

Supplement: Supplementary file 3 [file Image_1.jpeg]

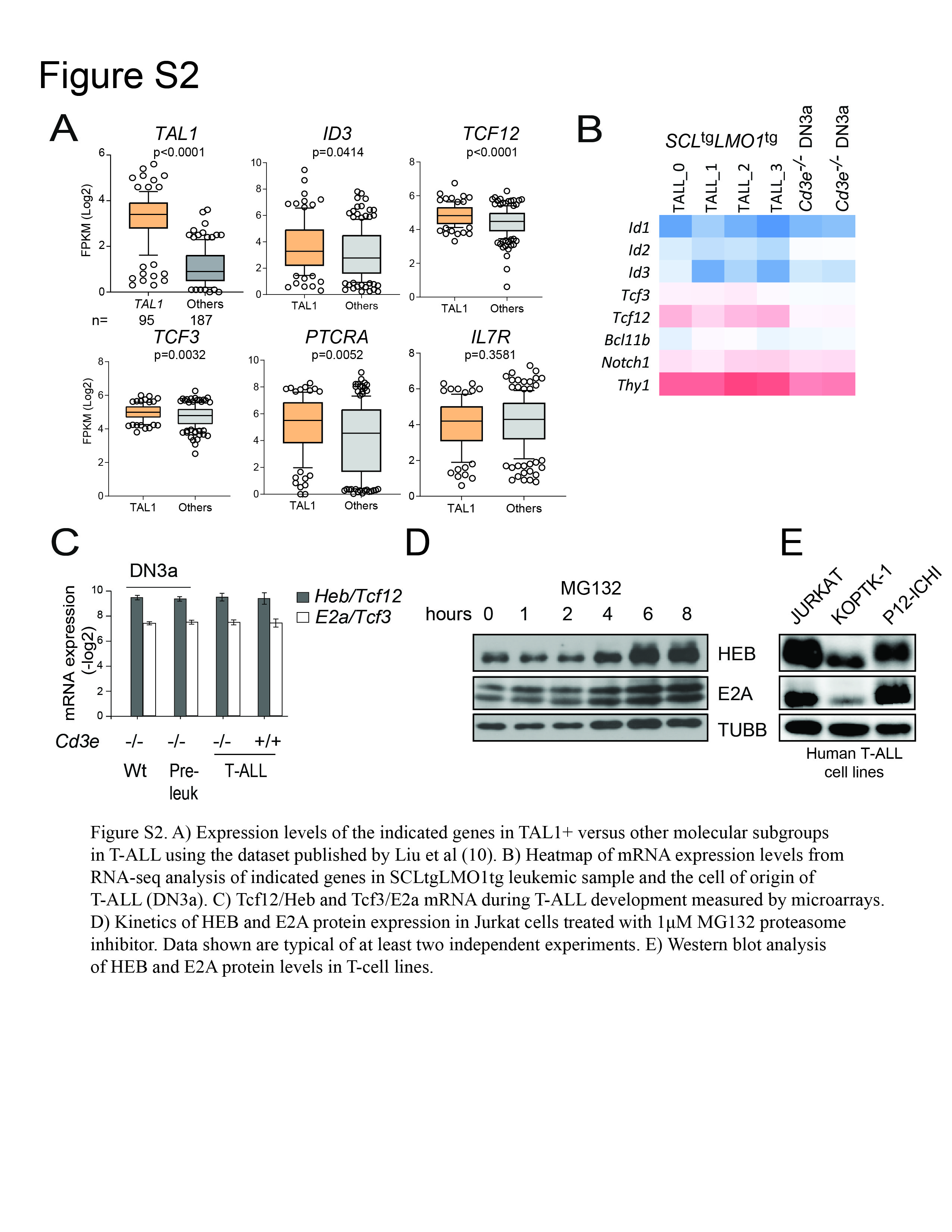

Supplement: Supplementary file 4 [file Image_2.jpeg]

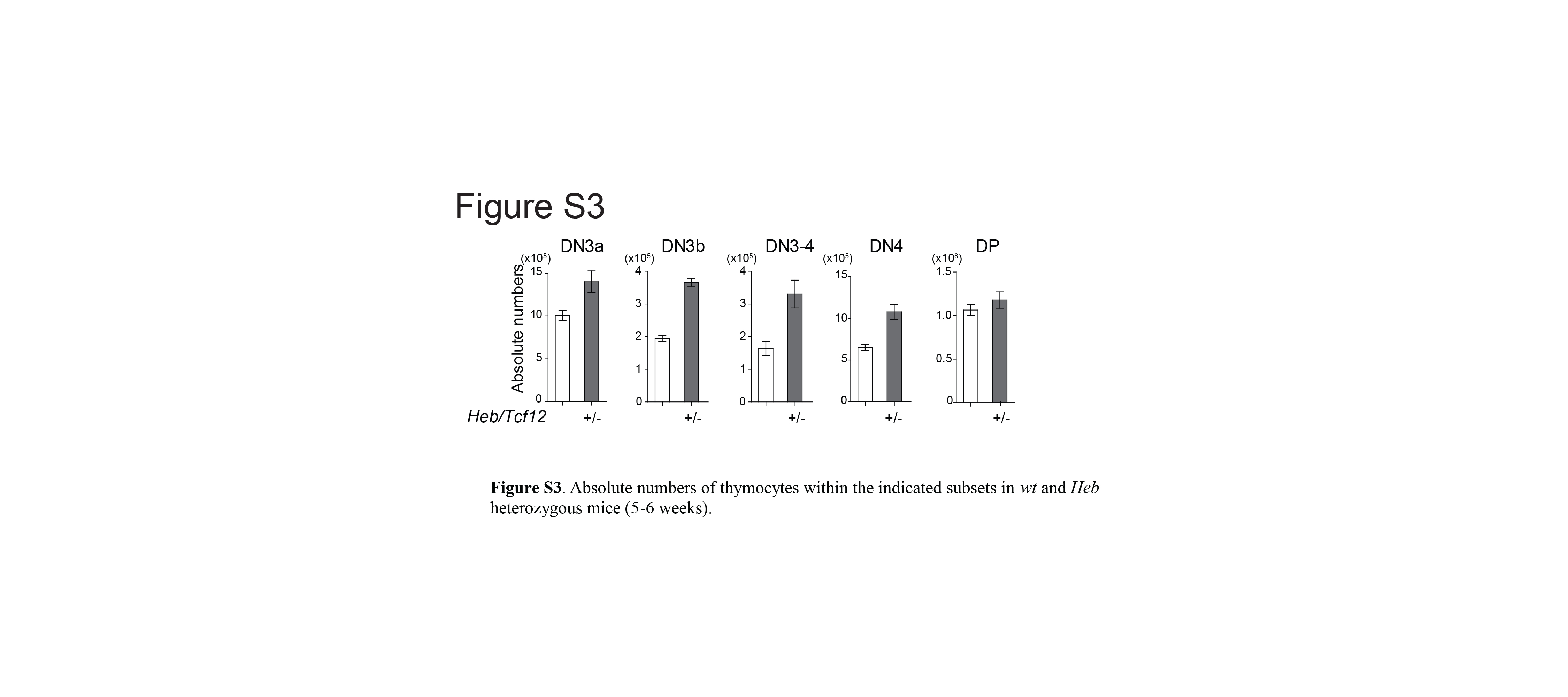

Supplement: Supplementary file 5 [file Image_3.jpeg]

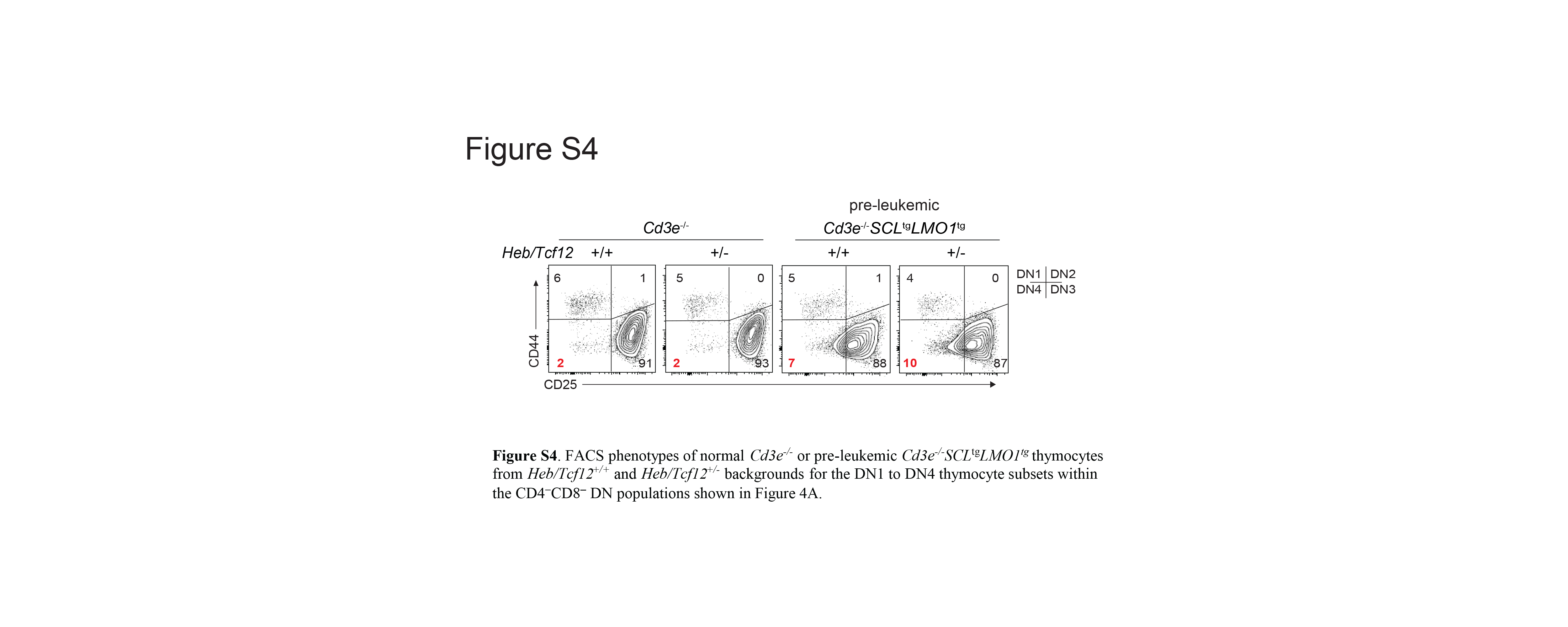

Supplement: Supplementary file 6 [file Image_4.jpeg]

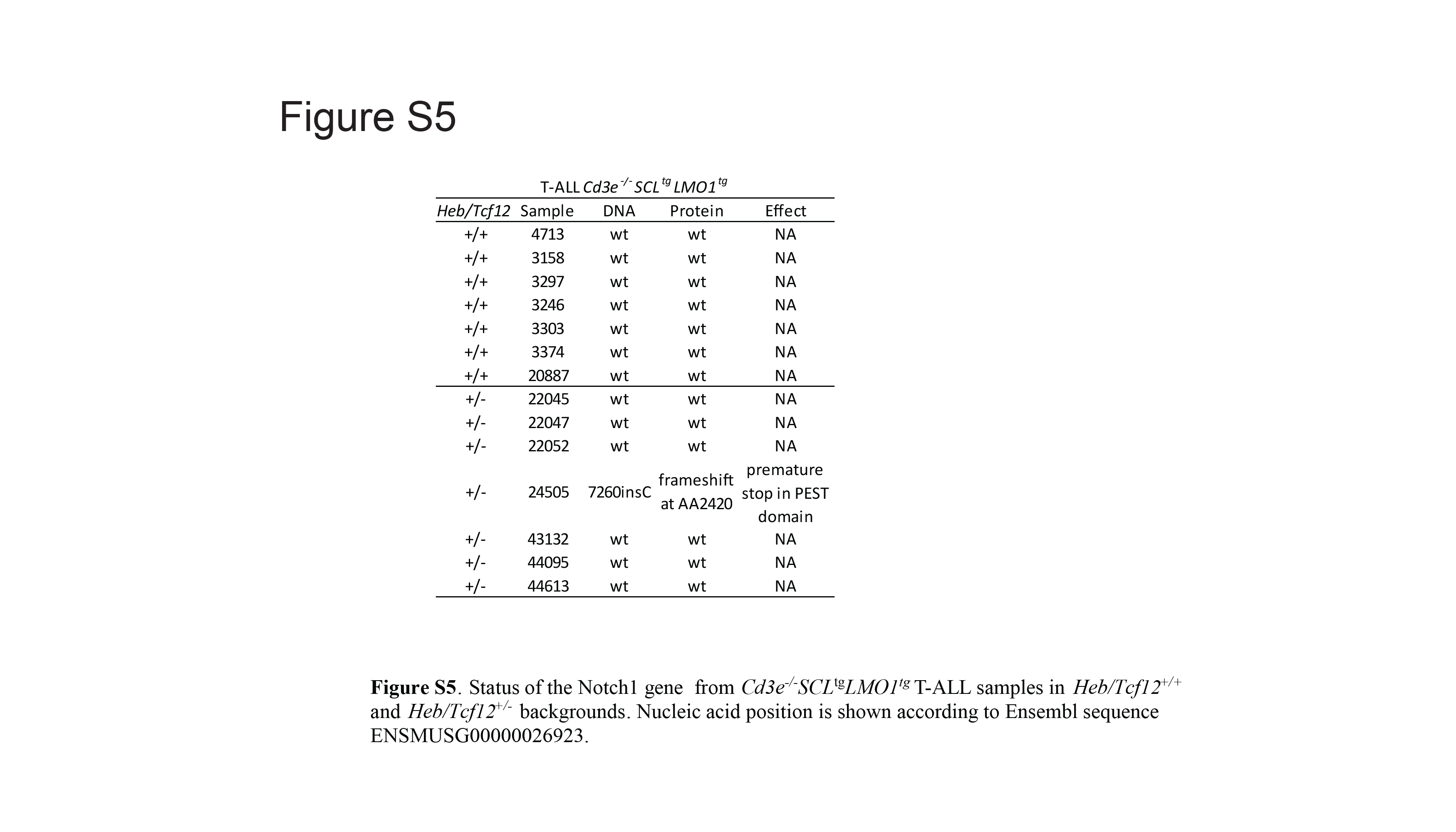

Supplement: Supplementary file 7 [file Image_5.jpeg]

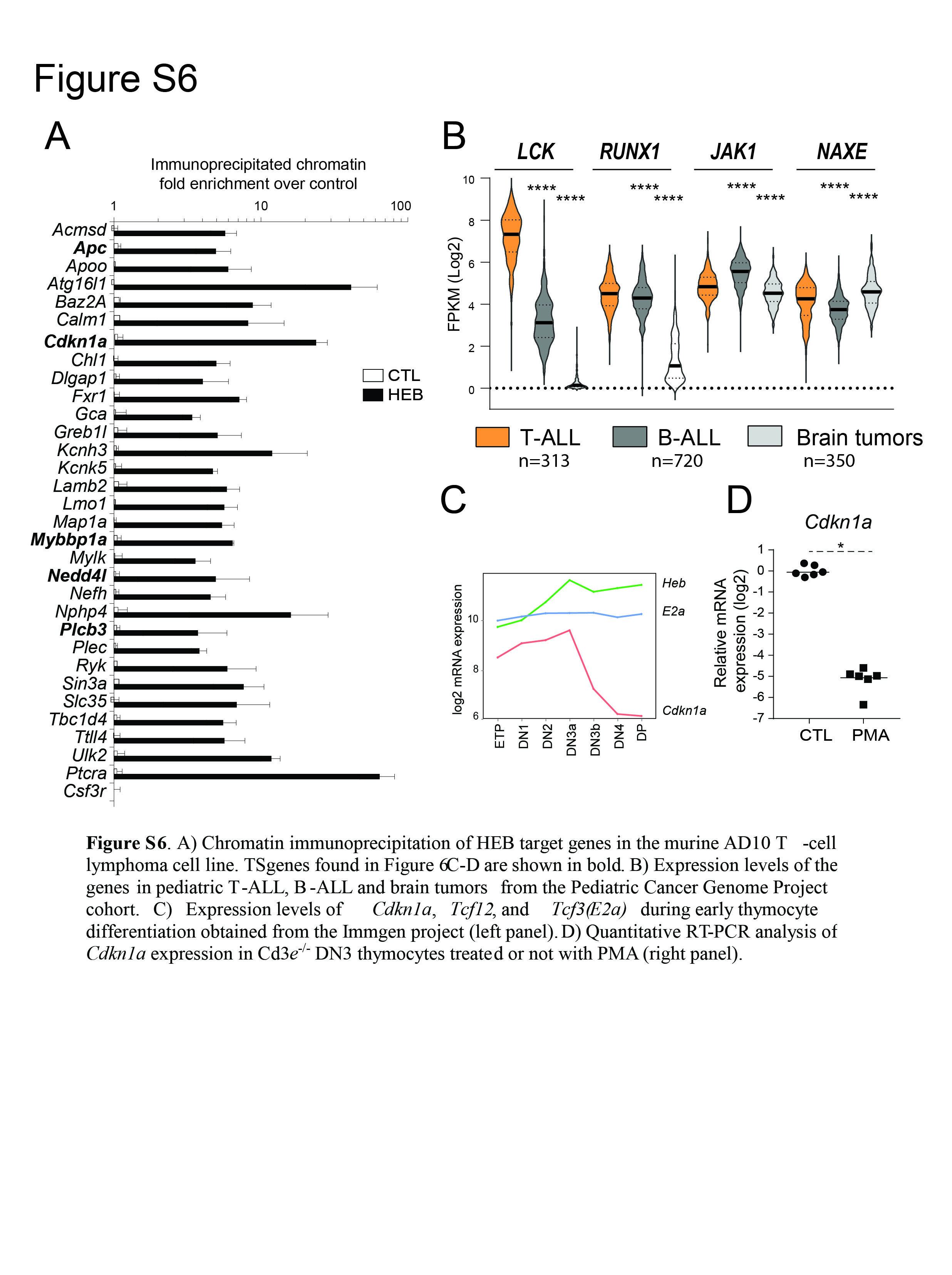

Supplement: Supplementary file 8 [file Image_6.jpeg]
